# Prioritising health research in KwaZulu-Natal: has the research conducted met the research needs?

**DOI:** 10.1186/s12961-020-0538-7

**Published:** 2020-03-18

**Authors:** G. Khumalo, R. Desai, X. Xaba, M. Moshabela, S. Essack, E. Lutge

**Affiliations:** 1 KwaZulu-Natal Department of Health, Health Research & Knowledge Management Unit, 330 Langalibalele Street, Pietermaritzburg, South Africa; 2 School of Nursing and Public Health, University of KwaZulu-Natal, 238 Mazisi Kunene Road, Glenwood, Durban, South Africa; 3 School of Health Sciences, University of KwaZulu-Natal, 238 Mazisi Kunene Road, Glenwood, Durban, South Africa

**Keywords:** research prioritisation, research priorities, prioritising research, priority research setting

## Abstract

**Background:**

The KwaZulu-Natal (KZN) Health Act of 2009 mandates the Provincial Health Research and Ethics Committee to develop health research priorities for the province. During 2013, the KZN Department of Health embarked on a research prioritisation process for the province. Priority research questions were generated by an inclusive process, in which a variety of stakeholders in health research in the province were engaged. The aim of this study was to determine whether research conducted at public health facilities in KZN between 01 January 2014 and 31 March 2017 met the research priorities of the province developed through the provincial research prioritisation process of 2013.

**Methods:**

This was a mixed methods study. Qualitative thematic analysis was used to categorise priority research questions generated in the priority-setting process and the titles of research projects conducted after that process into themes. Quantitative analysis was used to determine the correlation between themes of the priority questions, and those of the research projects conducted after the prioritisation exercise. Statistical Package for Social Science version 25 was used to analyse the data.

**Results:**

In 72% of thematic areas, there were disproportionately more priority questions than there were research projects conducted. There is thus a large disjuncture between the priorities developed through the provincial research prioritisation process of 2013 and the research projects conducted after that process in terms of major research areas.

**Conclusions:**

Ensuring that research conducted responds to priority questions raised is important because it ensures that research responds to locally important issues and to the concerns of local actors. Local health managers, communities and researchers should work together to ensure that the research conducted in their areas respond to the research priorities of those areas. Health Research Committees and local ethics committees can play important roles in facilitating the responsiveness to research priorities.

## Background

Research is recognised as an important tool to improve health and healthcare in South Africa. It ranks as one of the priorities of the National Department of Health (NDoH) in its ten-point plan [1] and is one of the key interventions of the KwaZulu-Natal (KZN) Provincial Growth and Development Plan [2]. However, because resources for research are limited, it is crucial to ensure that research is relevant for its target population.

The National Health Research Committee is tasked through the National Health Act (61 of 2003) with developing a national strategy for health research, ensuring that “*health research agendas and research resources focus on priority health problem*” ([3], p. 73). The NDoH held a national health research prioritisation exercise in 2011. Previous exercises had been held in 1996 and 2006. However, the effects of these research prioritisation exercises have not, to our knowledge, been evaluated. Specifically, the extent to which research conducted after such prioritisation answers the priority research questions identified has not been investigated.

In KZN, the KZN Health Act (2009) requires that the Provincial Health Research and Ethics Committee sets research priorities for the province and, accordingly, a research prioritisation exercise was conducted in 2013 [4]. A total of four workshops were conducted across the province, with clinicians, researchers and academics from a variety of organisations as well as representatives from non-governmental, community- and faith-based organisations. A total of 1018 priority research questions were identified, communicated to the leaders of all academic and research organisations in KZN, and posted on the KZN Department of Health (KZN-DoH) website to encourage the answering of these priority questions through research projects.

The main aim of the study was to determine the extent to which research conducted at public health facilities in KZN between 01 January 2014 and 31 March 2017 has met the research priorities of the province as developed in the research prioritisation process held in 2013. There were three objectives of the study. The first was to categorise the research questions developed during the prioritisation process of 2013 into thematic areas. The second was to categorise the research projects conducted between 01 January 2014 and 31 March 2017 into thematic areas, and the third was to investigate the level of concordance between the thematic areas of the priority research questions and those of the research projects conducted after the prioritisation process.

## Methods

### Data collection

The titles of research projects that had been conducted in the KZN public health facilities were obtained from the database of the Health Research and Knowledge Management Unit (HRKMU) of the KZN-DoH, which provides the provincial approval for all research conducted at provincial health facilities in the province. The priority research questions, as articulated through the research prioritisation process of 2013, were available from the KZN-DoH website.

### Data analysis

The titles of research projects conducted, and the priority research questions, were organised by three teams of investigators into themes. Each team consisted of two investigators who were paired to code both questions and titles according to deductive themes and subthemes guided by the broad Burden of Disease [5] and the WHO framework [6], as seen in Table 1. Then, each pair swapped their coding so that the next pair would code the same questions and titles. Whenever there were disagreements on the coding, a meeting was held to find consensus on coding. After the consensus meeting, themes and subthemes were finalised.

**Table 1:** Two sets of themes developed

Themes relating to burden of disease	Themes relating to the building blocks of a health system
Maternal and child health	Service delivery
Teenage pregnancy	Quality of care
Sexual and reproductive health	Health system
Tuberculosis	Infection prevention and control
HIV	Human resources management
Adherence (to treatment)	Information system
Non-communicable diseases, including injuries	Traditional medicine
Mental health	Clinical management and leadership/governance
Substance use	Health policy

Each title and research question was allocated two themes (a primary theme and a secondary theme) from either category. A grid structured according to these themes was developed (Appendix 1). Each title or question was coded first according to the primary theme and then according to the secondary theme. Thereafter, each coded title was placed into a specific cell in the grid for research titles, and each coded research question was allocated to a specific cell in the grid for research questions. This resulted in two grids – one for research titles, the other for research questions. The extent to which these two grids overlapped was then statistically assessed.

This statistical analysis was done in SPSS version 25. Each cell on each grid was allocated an SPSS code, which was a combination of the theme on the x axis (primary theme) and the theme on the y axis (secondary theme). For example, if the primary theme was ‘HIV’ and the secondary theme was ‘Quality of Care’, then the final SPSS code was ‘HIV/Quality of Care’. The frequency of the matching SPSS codes between the questions and titles was used to determine the extent to which priority research questions were answered by research projects.

## Results

A total of 1018 priority research questions and 1235 research titles were categorised into 32 primary and secondary themes. Table 2 shows the frequency of priority questions and project titles in each theme. Where the frequency is higher for the priority questions than that of the titles, this indicates that research needs were identified but not met. Where the frequency is higher for the project titles, this indicates that more research was conducted on a theme than was considered important during the prioritisation process. Where the frequencies are the same, this means that the research need identified through the prioritisation process was met by the research projects conducted. Overall, in 23 of the 32 themes developed, there was a higher frequency of research questions than titles, indicating that more questions were asked around this theme than research projects were conducted.

**Table 2:** Frequency showing research area needs that were met and unmet

	Questions	Titles	Titles/Questions (%)^a^
Adherence	2.6	1.3	0.50
Clinical management	4.4	18.4	4.18
Community health	5.6	1	0.18
Epidemiology	2.2	13.5	6.14
Evaluation/impact	2.4	1.1	0.46
Geography	1.8	0.4	0.22
Health economics	2.1	0.5	0.24
Health policy	1.2	1.2	1.00
Health promotion	3	0.7	0.23
Health systems	9.6	4.1	0.43
HIV/AIDS	8.3	4.2	0.51
Human resources management	10.1	10	0.99
Infection prevention and control	0.5	0.8	1.6
Infectious/communicable disease	1.2	1.6	1.33
Information systems	5.2	4.1	0.79
Intersectoral collaboration	3.1	0.4	0.13
Inter-sectoral collaboration, traditional	0.5	0	0.00
Malaria	1.7	0.4	0.24
Maternal and child health	8.2	5.8	0.71
Mental health	1.4	2.2	1.57
Non-communicable diseases	0.1	9.2	92.00
Nutrition	0	0.6	0.00
Occupational health and safety	1	1	1.00
Pregnancy-related healthcare	2.2	1.1	0.50
Quality of care	3.1	2.4	0.77
Service delivery	3.4	2.5	0.74
Sexual and reproductive health	1.4	1.5	1.07
Socio-behavioural and cultural factors	7.5	5.1	0.68
Substance use	0.1	0	0.00
Tuberculosis	4.1	3.9	0.95
Teenage pregnancy	0.7	0.5	0.71
Traditional medicine	1.3	0.5	0.38
Total	**100**	**100**	23/32 (72%) higher frequency of questions than titles

^a^ <1 indicates a research area need not met

**Fig. 1:**
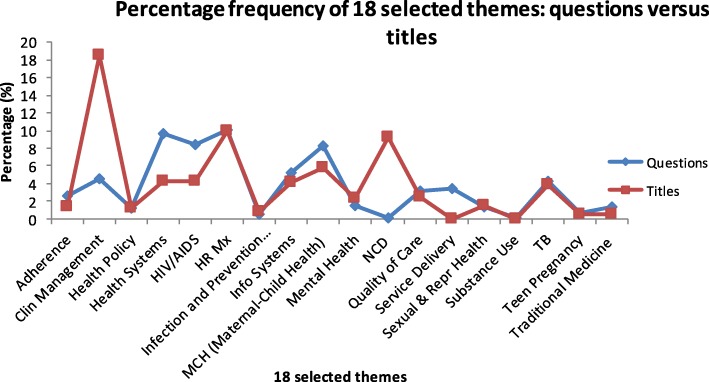
Percentage frequency of primary themes for the research questions and research titles

Figure 1 shows the frequency of primary themes for the research questions and research titles, where the percentage of each theme represents the relative number of times that theme occurred within the questions and titles, respectively. This figure illustrates the relative discrepancies between the primary themes of the priority research questions and the subsequent research projects.

As seen in Table 2 and Fig. 1, there was frequency discrepancy between conducted research and local research prioritisation questions. The themes that had no research conducted on them were Intersectoral collaboration, traditional, and Substance use. As seen in Table 3, 0.5% of the questions for Intersectoral collaboration, traditional, were on Geography and Health systems and HIV/AIDS and Traditional medicine were secondary themes. However, no research was conducted on Intersectoral collaboration during this study period. On Substance use, 0.1% of the questions were on the Community health as a secondary theme but no research was conducted on Substance use.

**Table 3:** Under researched health theme – Intersectoral collaboration, traditional

	Theme: Intersectoral collaboration, traditional	
	Questions	Titles
Geography	0.1	0
Health systems	0.2	0
HIV/AIDS	0.1	0
Traditional medicine	0.1	0
Total	0.5	0

Other under researched themes were Geography, Health promotion and Malaria, where research conducted was almost 0.2 times less that the research needed by the Province.

The themes that were mostly researched were Non-communicable diseases (NCDs) followed by Epidemiology and lastly Clinical management.

As seen in Table 4, on the NCD theme, the research prioritisation process questions had 0.1% of questions around the NCDs all of which were under the Community health secondary theme. However, research conducted on NCDs was 9.2%, which is 92 more times than what the Province indicated. Research was conducted mostly around Epidemiology (4.4.%) and Evaluation/impact (4.1%) secondary themes.

**Table 4:** Over researched health theme: Non-communicable diseases

	Non-communicable diseases	
	Questions	Titles
Adherence	0	0.2
Community health	0.1	0.1
Epidemiology	0	4.4
Evaluation/impact	0	4.1
Health systems	0	0.2
Human resources management	0	0.4
Total	**0.1**	**9.2**

For Epidemiology, six times more research (13.5/2.2) was conducted than what was indicated by the province (Table 5). Mostly the province indicated a need around Evaluation/impact (0.9%) and Nutrition (0.6%) secondary themes. However, no research was conducted around these secondary themes but instead research was conducted mostly around NCDs (5.3%) and HIV/AIDS (3.6%) as secondary themes.

**Table 5:** Over researched health theme: Epidemiology

	Epidemiology	
	Questions	Titles
Clinical management	0.3	0.3
Community health	0.1	0
Evaluation/impact	0.9	0
Health promotion	0	0.1
HIV/AIDS	0	3.6
Human resources management	0	0.1
Intersectoral collaboration	0.1	0
Maternal and child health	0	2.8
Non-communicable diseases	0	5.3
Nutrition	0.6	0
Pregnancy-related healthcare	0	1.1
Socio-behavioural and cultural factors	0.1	0
Substance use	0.1	0.2
Teen pregnancy	0	0.1
Total	2.2	13.5

For Clinical management, four times more research was conducted in the Province related to priority research questions (18.4%/4.4%) as seen in Table 6. For example, looking at the Evaluation/Impact secondary theme, 2% of priority questions were on this theme compared to 3.6% of research projects that were conducted around this theme.

**Table 6:** Over researched health theme: Clinical management

Clinical management		
	Questions	Titles
Evaluation/impact	2	3.6
Health systems	0.7	0.7
HIV/AIDS	0.9	2.6
Human resources management	0	0.6
Information systems	0.1	0
Maternal and child health	0.3	2.2
Mental health	0.2	0
Non-communicable diseases	0.2	6.2
Pregnancy-related healthcare	0	0.9
Sexual and reproductive health	0	0.2
Socio-behavioural and cultural factors	0.1	0.1
Tuberculosis	0	1.3
Teenage pregnancy	0	0.1
Total (percentage)	4.4	18.4

Some themes occurred with a similar frequency for research questions and titles. For example, Sexual and reproductive health had 1.4% research questions versus 1.5% titles. However, differences occurred in the secondary themes as seen in Table 7 regarding the specific aspects of sexual and reproductive health that were identified as priority research questions and that were the focus of research projects. Here, no priority questions looked at the epidemiology of sexual and reproductive health, and yet this was the focus of 0.3% of research projects. Similarly, whilst 0.4% of priority questions focused on teenage pregnancy within the theme of sexual and reproductive health, no research projects investigated this issue.

**Table 7:** Comparison of secondary themes under the primary theme of Sexual and reproductive health

	Questions	Titles
	Sexual and reproductive health (%)	
Community health	0	0.1
Epidemiology	0	0.3
Evaluation/impact	0.7	0.4
Health systems	0.2	0
Human resources management	0.1	0.4
Maternal and child health	0	0.2
Teenage pregnancy	0.4	0
Traditional medicine	0	0.1
Total (percentage)	**1.4**	**1.5**

Such detailed identification of the mismatch between research questions and research titles can be seen for the remaining themes in Appendix 2.

## Discussion

This study is, to our knowledge, the first to evaluate the implementation of a health research prioritisation exercise, in that it investigated the extent to which research projects focused on the issues raised in a research prioritisation exercise. Whilst a number of papers have considered the health research prioritisation process itself [7–12], none to our knowledge have looked at the effectiveness of such exercises. The evaluation of the implementation of research prioritisation exercises is important, because failure to conduct research on the priorities identified renders the prioritisation process useless and the resources spent on it wasted.

The study has shown that, following the identification of priority health research questions in KZN, the research projects conducted responded to the questions asked to varying degrees. Whilst the frequency of some primary themes are very similar in the priority questions and the research titles, in others, there are important differences, indicating that either priority questions were raised but were not addressed in research projects or that, despite not being identified as priority areas, research projects were conducted around certain themes. Even where the frequency of primary themes was similar across questions and titles, the frequency of secondary themes often showed discrepancies.

There is a discrepancy between local research priority and actual research conducted where one is either more or less than the other. This can be seen, for example, in the NCD theme, where more research was conducted than the prioritisation questions, as well as in the Intersectoral collaboration, traditional, theme, where no research was conducted even though this theme was indicated as a local priority during the prioritisation process. This reflects the degree of attractiveness of certain health areas where some are more attractive than the others. When weighing this attractiveness, it seems that either both funders and researchers are naïve of the research priority areas of the province or, if they are conscious about it, chose to ignore it. Prior to conducting research, researchers should self-reflect on what and how to prioritise research topics particularly in this era of “*decolonizing*” and “*participatory/embedded*” research [13]. Researchers play an important role in weighing the balance between global and local research interests, and finding ways of supporting locally relevant research drawn from local research prioritisation processes.

Ensuring that the research conducted responds to the priority questions raised is important because it ensures that research responds to locally important issues, and to the concerns of local actors. Both of these make it more likely that the results of research will be used in policy and practice. It is clear that researchers’ response to the KZN Research prioritisation process of 2013 was variable. The KZN HRKMU did not have the resources to fund research on priority questions, nor did HRKMU consider refusing permission for any research that did not respond to a priority question. We regard the latter as restrictive, because we acknowledge that research is a creative process and an important part of academic freedom and ‘scholarly debate’ [14].

## Conclusion

This assessment of the effectiveness of a research prioritisation process was one of the first studies of its kind. It shows that under one-third of the themes of priority questions developed in the KZN research prioritisation process were reflected in subsequent research projects. Thus, many areas of health and healthcare considered as priorities in the province remain under-researched. The province relies on evidence found through research to inform and improve the health system. It is hoped that subsequent research priorities will be met by research projects in the future, whilst maintaining the balance between freedom of academic enquiry and rooting research in agreed areas of priority.

## Recommendations

As the burden of disease in South Africa shifts and health issues take different levels of priority, research prioritisation processes can play an important role in directing local health research. The communication of research priorities resulting from these processes should be enhanced, and local funding made available, to encourage studies that focus on priority research questions. Local health managers, communities and researchers should work together to ensure that the research conducted in their areas responds to the research priorities of those areas. This will require the strengthening of relationships at all levels. Provincial Health Research Committees and local ethics committees can play important roles in facilitating this.

## Data Availability

The datasets used and/or analysed during the current study are available from the corresponding author on reasonable request.

## Appendix 1

**Table 8:** Grid to show distribution of research titles/questions according to thematic area

	Adherence	Clinical management	Community health/community-based healthcare	Epidemiology	Impact/evaluation	Geography	Health economics	Health policy	Etc.
Adherence									
Clinical management									
Community health/community-based healthcare									
Epidemiology									
Evaluation/impact									
Geography									
Health economics/finance									
Health policy and guidelines									
Health promotion									
Health systems									
HIV/AIDS									
Human resources/management									
Infection prevention and control									
Infectious/communicable diseases									
Information systems/informatics									
Intersectoral collaboration									
Intersectoral collaboration, traditional									
Malaria									
Maternal and child health									
Mental health									
Non-communicable diseases									
Nutrition									
Occupational health and safety									
Pregnancy-related healthcare									
Quality of care									
Service delivery									
Sexual and reproductive health									
Socio-behavioural and cultural factors									
Substance use									
Tuberculosis									
Teenage pregnancy									
Traditional medicine									

## Appendix 2

**Table 9:** Matching between research questions and research titles

	Questions	Titles	Questions	Titles	Questions	Titles	Questions	Titles	Questions	Titles	Questions	Titles	Questions	Titles	Questions	Titles	Questions	Titles
	Clinical management		Non-communicable diseases		Sexual and reproductive health		Mental health		Infection prevention and control		Health policy		Epidemiology		Health policy		Nutrition	
Adherence				0.2														
Clinical management								0.3					0.3	0.3				0.2
Community health			0.1	0.1		0.1		0.1					0.1					
Epidemiology				4.4		0.3	0.2	1.1										
Evaluation/impact	2	3.6		4.1	0.7	0.4	0.4	0.3	0.7	0.6	0.7	0.6	0.9		0.7	0.6		0.4
Health promotion								0.2						0.1				
Health systems	0.7	0.7		0.2	0.2		0.6		0.2	0.5	0.2	0.5			0.2	0.5		
HIV/AIDS	0.9	2.6						0.1						3.6				
Human resources management		0.6		0.4	0.1	0.4			0.2		0.2			0.1	0.2			
Info systems	0.1	0					0.1											
Intersectoral collaboration													0.1					
Maternal and child health	0.3	2.2				0.2								2.8				
Mental health	0.2																	
Non-communicable diseases	0.2	6.2												5.3				
Nutrition													0.6					
Pregnancy-related healthcare		0.9								0.1		0.1		1.1		0.1		
Quality of care																		
Sexual and reproductive health		0.2							0.1		0.1				0.1			
Socio-behavioural and cultural factors	0.1	0.1					0.2	0.2					0.1					
Substance use													0.1	0.2				
Tuberculosis		1.3																
Teenage pregnancy		0.1			0.4									0.1				
Traditional medicine						0.1												
Total	**4.4**	**18.4**	**0.1**	**9.2**	**1.4**	**1.5**	**1.4**	**2.2**	**0.5**	**0.8**	**1.2**	**1.2**	**2.2**	**13.5**	**1.2**	**1.2**	**0**	**0.6**

## Abbreviations

HRKMU: Health Research and Knowledge Management Unit
IPC: Infection Prevention and Control
KZN: KwaZulu-Natal
KZN-DoH: KwaZulu-Natal Department of Health
NCDs: non-communicable diseases
NDoH: National Department of Health
